# Increased creatinine clearance in polytrauma patients with normal serum creatinine: a retrospective observational study

**DOI:** 10.1186/cc10013

**Published:** 2011-02-03

**Authors:** Vincent Minville, Karim Asehnoune, Stephanie Ruiz, Audrey Breden, Bernard Georges, Thierry Seguin, Ivan Tack, Acil Jaafar, Sylvie Saivin, Olivier Fourcade, Kamran Samii, Jean Marie Conil

**Affiliations:** 1 Department of Anesthesiology and Intensive Care, GRCB 48, IFR 150, Toulouse University Hospital, Toulouse, France; 2 Department of Anesthesiology and Surgical Intensive Care Medicine, Hôtel Dieu Hôpital Mère Enfant, C.H.U Nantes, France; 3 Service d'Explorations Fonctionnelles Physiologiques, INSERM U858, IFR150, Hôpital de Rangueil, 31403 Toulouse, France; 4 Laboratoire de Pharmacocinétique et Toxicologie Clinique, Institut Fédératif de Biologie, 330 Avenue de Grande Bretagne, TSA 40031, 31059 Toulouse Cedex 9, France

## Abstract

**Introduction:**

The aim of this study, performed in an intensive care unit (ICU) population with a normal serum creatinine, was to estimate urinary creatinine clearance (CL_CR_) in a population of polytrauma patients (PT) through a comparison with a population of non trauma patients (NPT).

**Methods:**

This was a retrospective, observational study in a medical and surgical ICU in a university hospital. A total of 284 patients were consecutively included. Two different groups were studied: PT (*n *= 144) and NPT (*n *= 140). Within the second week after admission to the ICU, renal function was assessed using serum creatinine, 24 h urinary CL_CR _.

**Results:**

Among the 106 patients with a CL_CR _above 120 mL minute^-1 ^1.73 m^-2^, 79 were PT and 27 NPT (*P *< 0.0001). Only 63 patients had a CL_CR _below 60 mL minute^-1 ^1.73 m^-2 ^with 15 PT and 48 NPT (*P *< 0.0001). Patients with CL_CR _greater than 120 mL minute^-1^. 1.73 m ^-2 ^were younger, had a lower SAPS II score and a higher male ratio as compared to those having CL_CR _lower than 120 mL minute^-1^. 1.73 m ^-2^. Through a logistic regression analysis, age and trauma were the only factors independently correlated to CL_CR_.

**Conclusions:**

In ICU patients with normal serum creatinine, CL_CR_, is higher in PT than in NPT. The measure of CL_CR _should be proposed as routine for PT patients in order to adjust dose regimen, especially for drugs with renal elimination.

## Introduction

Early detection of renal dysfunction in intensive care unit (ICU) patients is important. Indeed, an increase of the glomerular filtration rate (GFR) was demonstrated in some ICU patients populations by using urinary creatinine clearance (CL_CR_) as a surrogate marker, and many of the drugs used in ICU patients need dose adjustment as a function of GFR [1]. Despite a normal serum creatinine measurement, a substantial number of burn patients demonstrated an increase in GFR with the need for increasing doses of renal elimination drugs to maintain therapeutic concentrations [1,2]. A study of our group showed that 42% of burn patients had a creatinine clearance greater than 120 mL minute^-1 ^1.73 m^2 ^[1]. Also, several studies performed in a general population of ICU patients suggested a poor correlation between serum creatinine concentration and GFR in polytrauma patients (PT) [3-5]. To the best of our knowledge, no study has specifically explored this population of PT patients.

The aim of this study, performed in a population of ICU patients with normal serum creatinine, was to estimate GFR, evaluated by measured CL_CR_, in a population of PT patients through a comparison with a population of non-trauma patients (NPT).

## Materials and methods

### Patients

This observational study was conducted in the ICU of Toulouse University hospital during a five-year period (November 2002 to December 2007). The study was performed according to the Declaration of Helsinki. No change in our current clinical practice (measured creatinine clearance monitoring, at least once a week, is a part of the routine medical care of the patients) and no randomization was performed. As it was an observational retrospective study, in accordance with French law, neither approval of the ethics committee nor informed consent was required.

Ten days, on average, after admission in ICU, consecutive critically ill patients meeting the inclusion criteria were included. Inclusion criteria were: patients older than 18 years, with an arterial catheter, a urinary bladder catheter, a diuresis over 500 mL d^-1^. All patients had a tracheal tube and were mechanically ventilated. Patients were hemodynamically stable presenting with a stable serum creatinine in a normal range (40 to 125 μmol L^-1^).

Patients were excluded from the study: if they were hemodynamically unstable or if they needed a high dose of catecholamine (norepinephrine > 1 mg h^-1^); if they were recovering from acute kidney injury (AKI) or developing AKI; if they received histamine-2-receptor antagonist due to its interference with tubular creatinine secretion [6]; and if they had a medical history of diabetes, of chronic hepatic disease, cirrhosis or ongoing liver dysfunction with hepatitis [7,8]. Patients treated with diuretics were also excluded.

Baseline characteristics of patients were recorded at enrolment in the study and the SAPS II was obtained at ICU admission. PT patients had an ISS (Injury Severity Score) > 16. SOFA (Sequential Organ Failure Assessment) score was obtained on the day the urine 24-hour measure was sampled [9-11].

Urine was sampled over 24 hours to measure urinary creatinine concentration. Serum creatinine was also measured during the urine collection period.

The normal limits of CL_CR _were estimated between 60 and 120 mL minute^-1 ^1.73 m^-2 ^[9,12].

### Serum creatinine measurement and calibration

Creatinine measurements were performed in the same laboratory of the University Hospital of Toulouse. Blood samples were obtained simultaneously with the CL_CR _measurement. A modified kinetic Jaffe colorimetric method was used with a COBAS MIRA (ABX Diagnostics, Montpellier, France) analyzer. A two-point calibration was applied in each assay.

Before measurement, ultrafiltration of plasma through a 20 kD cutoff membrane (MPS-1; Amicon, Beverly, MA, USA) was performed to discard chromogens that were linked to albumin-like bilirubinemia and other heavy proteins. In the absence of an international standard for creatinine assay, the linearity of the measurements was verified by using plasma samples from normal subjects in which increasing amounts of desiccated creatinine hydrochloride (MW 149.6; Sigma Chemicals, Perth, Australia) had been added. Linear regression analysis showed that the relationship between measured and expected creatinine concentrations was 1.0008 ± 0.006 (95% confidence interval, 0.997 to 1.020) and that the Y-intercept was 0.014 ± 0.013 (95% confidence interval, -0.013 to 0.041). Squared Spearman rank coefficient of correlation was 0.998. Internal quality controls showed a coefficient of variation of 2.3% during the period.

### Assessment of glomerular filtration rate

Creatinine clearance was measured according to the formula $CL_{CR}=\frac{UC_{R}\times V}{SC_{R}}$ where urine creatinine (UCR) and serum creatinine (SCR) were expressed in μmol L^-1 ^and V corresponded to the urinary rate (diuresis) in mL minute^-1^.

At the same time, the GFR was estimated using the Cockcroft Gault formula [13]$CL_{CR}=\frac{(140-age)\times Weight}{0.8\times SC_{R}}$ for men, with age in years and weight in Kg. A correcting factor of 0.85 was used for women. The derivate formula proposed by Robert *et al. *[14] uses the ideal body weight and serum creatinine concentration corrected to 85 μmol L^-1 ^when the actual value is lower than 85 μmol L^-1^. Ideal body weight was determined as 50 kg for men and 45.5 kg for women, plus 2.3 kg for each inch over five feet. The simplified formula of the Modification of Diet in Renal Disease index (sMDRD) [15] was also calculated according to sMDRD = 186.3 × SCR ^-1.154 ^× age^-0.203 ^× (1.212 if black) × (0.742 if female) where serum creatinine was expressed in mg dL^-1^.

### Statistical analysis

Statistical analyses were performed using StatView^® ^software version 5.0 (SAS Institute Inc., Cary, NC, USA). Data are presented as mean ± standard deviation (SD) or ratio. Normal distribution of data was tested via Kolmogorov-Smirnov test. Chi-square test or Student's *t*-test was performed when appropriate. A logistic regression was performed to discriminate if trauma, age, SAPS II, ideal body weight and sex are independently correlated to the measured CL_CR_. A *P*-value < 0.05 was considered as statistically significant.

## Results

**Figure 1:**
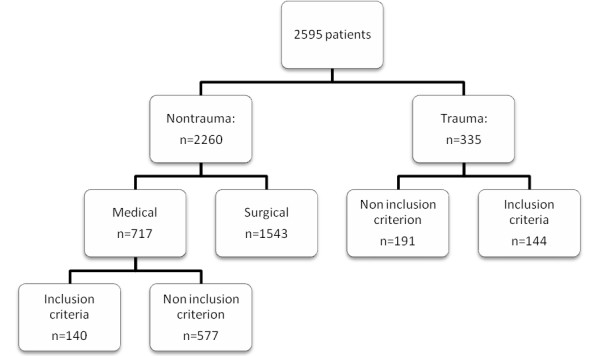
**Flow chart showing the process of recruitment**.

Demographic and renal data are shown in Table 1. Two hundred, eighty-four patients were consecutively included in this observational study. The process of screening and inclusion in the study is shown in Figure 1 (flow chart).

**Table 1:** Demographic data

	NPT^a ^(*n *= 140)	PT^b ^(*n *= 144)	P*
Age (yr)	58 ± 17	42 ± 18	< 0.0001
Weight (kg)	72 ± 18	75 ± 14	NS
Height (cm)	170 ± 8	174 ± 9	NS
Sex (F/M)	52/88	36/108	0.03
Ideal body weight (Kg)	68 ± 11	72 ± 11	0.0006
SAPS 2	52 ± 14	42 ± 15	< 0.0001
			
Total SOFA score	3.7 ± 1.5	3.6 ± 1.4	NS
Respiratory system	0.3 ± 0.2	0.5 ± 0.6	
Coagulation	0.0 ± 0.0	0.0 ± 0.0	
Liver	0.0 ± 0.0	0.0 ± 0.0	
Cardiovascular system	1.3 ± 0.9	1.4 ± 0.9	
Neurological system	2.5 ± 1.5	2.4 ± 1.5	
Renal system	0.0 ± 0.0	0.0 ± 0.0	
			
Mean arterial blood pressure (mmHg)	83 +/- 10	82 +/- 11	NS
Systolic arterial blood pressure (mmHg)	126 +/- 16	125 +/- 17	NS
Diastolic arterial blood pressure (mmHg)	63 +/- 10	63 +/- 11	NS
Heart rate (bpm)	91 +/- 17	92 +/- 16	NS
Serum creatinine (μmol L-1)	74 ± 26	72 ± 19	NS
Measured creatinine clearance (mL minute^-1 ^1.73 m-2)	85 ± 5	131 ± 5	< 0.0001
Diuresis/24H	2,700 ± 1,200	2,500 ± 1,200	NS
Serum Urea	8.7 ± 5	7 ± 3	NS
Measured creatinine clearance < 60 (mL minute^-1 ^1.73 m-2)	48	15	0.0003
Measured creatinine clearance > 120 (mL minute^-1 ^.1.73 m-2)	27	79	< 0.0001

All data are expressed as ratio or mean ± SD.

* The *P*-values correspond to comparison between NPT and PT groups.

^a ^NPT, non-polytrauma patients.

^b ^PT, polytrauma patients.

The group of 144 PT patients was compared with the group of 140 NPT patients. No difference was found concerning hemodynamic data (Table 1). No difference was found concerning ventilation pressure. All the patients were ventilated with a tidal volume of 6 to 8 ml/kg, the PEP value was set at 5.8 ± 3 in PT vs 5.5 ± 3 in NPT patients (NS). FiO2 was 45 +/- 16% in PT vs 45 +/- 15 in NPT (NS), with Ph = 7.38 +/- 0.8 vs 7.39 +/- 0.8 (NS), PaO2 = 107 +/- 16 in PT vs 108 +/- 15 in NPT (NS), PaCO2 = 39 +/- 8 in PT vs 40 +/- 9, SaO2 = 97 +/- 3 vs 97 +/- 3 (NS). Glycemia was not different between groups (6.2 +/- 1.7 vs 6.1 +/- 1.8; NS). Twenty-three percent of PT vs 24% of NPT received norepinephrine (NS).

The overall results show that serum creatinine was normal (73 ± 22 μmol L^-1^) and serum urea (8 ± 4 mmol L^-1^) was slightly higher than the normal limits, but with no difference between groups. One hundred, six patients had a CL_CR _above 120 mL minute^-1 ^1.73 m^-2^, including 79 PT and 27 NPT (*P *< 0.0001). Only 63 patients had a CL_CR _below 60 mL minute^-1 ^1.73 m^-2 ^with 15 PT and 48 NPT (*P *< 0.0001), whereas nine patients had a CL_CR _below 30 mL minute^-1 ^1.73 m^-2^, including two PT.

The overall urinary creatinine excretion was 929 ± 678 mg 24 h^-1 ^1.73 m^-2 ^for women and 1,369 ± 685 mg 24 h^-1 ^1.73 m^-2 ^for men. There was a significant difference between the urinary creatinine excretion of PT and NPT patients (1,489 ± 639 vs 969 ± 688 mg 24 h^-1 ^1.73 m^-2 ^respectively, *P *< 0.001). In the PT group, males had significantly higher urinary creatinine excretion than females (1,630 ± 644 vs 1,067 ± 392 mg 24 h^-1 ^1.73 m^-2^, *P *< 0.001).

The overall measured CL_CR _was 108 ± 57 mL minute^-1 ^1.73 m^-2^. The CL_CR _was higher in PT patients than in NPT patients when using measured CL_CR _(131 ± 56 vs 85 ± 48 mL minute^-1 ^1.73 m^-2 ^respectively, *P *< 0.001).

Most patients with increased CL_CR _(above 120 mL minute^-1 ^1.73 m^-2^) were PT patients as shown in Table 2. On the opposite, most patients with decreased CL_CR _(below 60 mL minute^-1 ^1.73 m^-2^) were NPT patients.

**Table 2:** Comparison of patients with different measured creatinine clearance (CL_CR_)

	Cl_CR _< 120 (*n *= 178)	Cl_CR _> 120 (*n *= 106)	P *	Cl_CR _< 60 (*n *= 63)	Cl_CR _> 60 (*n *= 221)	*P * ^#^	*P§*
Age (yr)	56 ± 18	40 ± 16	< 0.0001	63 ± 15	46 ± 18	< 0.0001	< 0.0001
Weight (Kg)	74 ± 17	74 ± 14	NS	73 ± 19	74 ± 15	NS	NS
Sex (F/M)	64/114	24/82	0.03	35/28	161/60	NS	0.0032
Ideal body weight (Kg)	69 ± 12	72 ± 10	0.01	65 ± 11	71 ± 11	0.0002	< 0.0001
SAPS 2	50 ± 15	43 ± 14	0.0002	54 ± 14	45 ± 15	< 0.0001	< 0.0001
Serum creatinine (μmol L^-1^)	76 ± 24	67 ± 19	0.0001	85 ± 25	69 ± 20	< 0.0001	< 0.0001
Diuresis/24H	2,400 ± 100	2,800 ± 1200	< 0.0001	584 ± 224	1,418 ± 694	0.0019	< 0.0001
Measured creatinine clearance (mL min^-1 ^.1.73 m^-2^)	74 ± 30	166 ± 50	< 0.0001	43 ± 11	127 ± 50	< 0.0001	< 0.0001
Simplified MDRD (mL minute^-1 ^1.73 m^-2^)	96 ± 39	119 ± 45	< 0.0001	81 ± 34	112 ± 43	< 0.0001	< 0.0001
Cockcroft-Gault (mL minute^-1 ^1.73 m^-2^)	98 ± 39	130 ± 34	0.0005	80 ± 39	118 ± 36	< 0.0001	< 0.0001
Robert (mL minute^-1 ^1.73 m^-2^)	72 ± 23	94 ± 23	< 0.0001	59 ± 2	85 ± 2	< 0.0001	< 0.0001
PT ^b^/NPT ^a^	65/113	79/27	< 0.0001	15/48	129/92	< 0.0001	< 0.0001

All data are expressed as ratio or mean ± SD.

* The *P*-values correspond to comparison between subgroup with CL_CR _greater than 120 mL minute^-1 ^1.73 m^-2 ^versus subgroup with CL_CR _lower than 120 mL minute^-1 ^1.73 m^-2^.

^# ^The *P*-values correspond to comparaison between subgroup with CL_CR _greater than 60 mL minute^-1 ^1.73 m^-2 ^versus subgroup with CL_CR _lower than 60 mL minute^-1 ^1.73 m^-2^.

§ The *P*-values correspond to comparison between less than 60 mL minute^-1 ^1.73 m^-2 ^more than 120 mL minute^-1 ^1.73 m^-2^.

^a ^NPT = non-polytrauma patients.

^b ^PT = polytrauma patients.

Patients with CL_CR _greater than 120 mL minute^-1 ^1.73 m ^-2 ^were younger (40 ± 16 years vs 56 ± 18 years), had a lower SAPS II score (43 ± 14 vs 50 ± 15) and a higher male ratio as compared with patients presenting a CL_CR _lower than 120 mL minute.

All factors presenting a statistical difference between hyperfiltration and hypofiltration subgroups (Table 2) were analyzed. Through a logistic regression analysis, including goodness of fit of the model, age and trauma were the only factors independently correlated to CL_CR _(Table 3).

**Table 3:** Logistic regression for different measured creatinine clearance

	P *	Odd ratio (CI 95%)
CL_CR _> 120 mL minute^-1 ^1.73 m^-2^		

Age	< 0.0001	0.95 (0.93 to 0.97)
SAPS 2	0.56	1.00 (0.98 to 1.04)
Ideal body weight	0.29	0.97 (0.93 to 1.02)
Gender	0.564	0.41 (0.12 to 1.4)
PT	0.0001	3.33 (1.8 to 6)

CL_CR _< 60 mL minute^-1 ^1.73 m^-2^		

Age	< 0.0001	0.95 (0.93 to 0.97)
SAPS 2	0.36	0.98 (0.95 to 1.02)
Ideal body weight	0.3	1.03 (0.97 to 1.09)
Gender	0.8	0.84 (0.97 to 1.09)
NPT	0.02	2.39 (1.15 to 4.97)

PT = polytrauma patients, NPT non polytrauma patients.

* The *P*-values correspond to logistic regression with CL_CR _greater than 120 mL minute^-1^1.73 m^-2 ^(C index = 0.56).

^# ^The *P*-values correspond to logistic regression with CL_CR _lower than 60 mL minute^-1 ^1.73 m^-2^

(C index = 0.58).

## Discussion

The present results comparing a population of hemodynamic stable PT patients to a population of hemodynamic stable NPT patients with steady state serum creatinine concentration with a normal creatinine serum value demonstrate that (i) PT patients exhibit dramatic variations of their CrCl; (ii) CrCl is higher in PT patients than in NPT patients; (iii) Age and trauma are independently correlated factors to CL_CR _in our study and in these study conditions.

Considering serum creatinine values, no significant difference was found between PT and NPT groups despite the variations of CrCl. These data demonstrated that a wide range of measured CL_CR _variations exists and, therefore, confirm Hoste data obtained in critically ill patients with serum creatinine within normal range [16]. These authors demonstrated that "serum creatinine has a low sensitivity for detection of renal dysfunction". Our results also revealed some opposite trends between CrCl and creatinine measurements, as some patients had significantly lower values of serum creatinine for CL_CR _> 60 mL minute^-1 ^1.73 m^-2 ^than for CL_CR _< 60 mL minute^-1 ^1.73 m^-2^. These data underline the inaccuracy of serum creatinine values in estimating the renal function.

Fifty-five percent of PT patients, and only 19% of NPT patients presented a measured CL_CR _above 120 mL minute^-1 ^1.73 m^-2^. In addition, only 10% of PT patients vs 34% of NPT patients presented a measured CL_CR _below 60 mL minute^-1 ^1.73 m^-2^. In clinical practice, the diagnosis of increased CL_CR _as a surrogate marker of GFR is important and has largely been demonstrated in burn patients in the setting of antibiotics monitoring: ceftazidime, cefepime, vancomycin and amikacin [4,5,17,18]. In critically ill patients, high CL_CR _required high doses of drugs, which are eliminated by the kidneys to obtain therapeutic concentration. Recently we confirmed the need for CL_CR _monitoring in order to accurately monitor renal function and, therefore, to optimize the doses of antibiotics [4,19].

Our results demonstrate that age, gender, ideal body weight, severity index, trauma patients, and serum creatinine are factors for a CL_CR _above normal (> 120 mL minute^-1 ^1.73 m^-2^), and for a moderate renal impairment (CL_CR _< 60 mL minute^-1 ^1.73 m^-2^). Through a logistic regression analysis, only two factors (age and trauma patients) remained significantly correlated with a CL_CR _above normal and for a moderate renal impairment. In the current results, 12% of elderly patients (over 65 years) have a CL_CR _greater than 120 mL minute^-1 ^1.73 m^-2^. The impact of age on CL_CR _is well known and this parameter was, therefore, introduced in the formulas estimating CL_CR _(Cockcroft-Gault, Robert and simplified MDRD) [13-15]. The decrease in glomerular filtration, the involution of nephronic units and the reduction of the renal blood flow explain the high frequency of renal impairement in elderly patients. However, it should be kept in mind that glomerular ageing is correlated to age in only two-thirds of the patients, and this phenomenon accounts for the inaccuracy of the CL_CR _estimated by calculated formulae [20].

Current evidence suggests that PT (mainly, young patients without significant comorbidities) present with a CL_CR _increase. However, this phenomenon as received little attention in the literature, and dose modification are therefore rarely considered. The present results clearly demonstrate for the first time that trauma is a major factor for CL_CR _increase. Several factors may explain this increase in CL_CR _in PT patients. First, urinary creatinine excretion may be involved in such a phenomenon. A higher creatinine urinary excretion was observed in PT compared with NPT patients whereas serum creatinine was similar in both groups. However, the higher creatinine urinary excretion observed in PT patients was within a normal range. Serum protein variations may impact our results. However, all the patients had a serum protein value between 50 and 55 gL^-1^. It is, therefore, very unlikely that serum protein variations interfere with the present results. Also, regarding hemodynamics, CL_CR _were studied and measured at a steady state in both groups (that is, distant from the admittance). It should be noted that our patients were hemodynamically stable at the time of data collection with no sign of dehydration. Although interference due to some cephalosporin has been described when the creatininemia was measured by using the Jaffe method [21]; no changes in this parameter were observed during the overall period of the study. Sepsis can also reduce creatinine production as described in mice [22]. Moreover, in critically-ill patients, a positive fluid balance may lead to underestimation of the severity of AKI and delay the recognition of a 50% relative increase in sCr [23]. Finally, it should be hypothesized that humoral and inflammatory mechanisms encountered after severe trauma [24] or burn [1] are involved in the observed CL_CR _increase.

The present study encountered some limitations. Increased CL_CR _is related with enhanced renal elimination of circulating drugs. However, describing this phenomenon in terms of current available measures of the GFR at the bedside is still debated. In our study, the GFR was estimated by measured creatinine clearance on a 24-hour urine collection. However, the gold standard for GFR assessment is the measure of inuline clearance [25] but the cost and complexity of this tool limits its application in routine. Another limitation is the lack of consensus regarding the upper limit of normal GFR. However, increasing data support the concept of increased GFR in PT patients, and several reports demonstrated subtherapeutic concentrations of drugs in PT patients [5,26]. Also, a cross-sectional single 24-hr measure of CL_CR _at 10 days in relatively stable patients was performed but fast modifications of kidney function may occur and there is a need for a continuous re-evaluation. Finally, some other factors may influence our results. In particular, fluid status, cardiac output may be significantly altered from baseline. However, whatever the causes of these alterations, the ATLS (Advanced Trauma Life support) principles are applied in our institution regarding resuscitation of PT patients. We, therefore, believe that the current results are broadly representative of the population of PT patients. It could be argued that the external validity of this single-center study may be limited. However, our findings may be relevant to the vast majority of level I trauma centers, provided that ATLS principles are applied in these institutions.

## Conclusions

In hemodynamic ICU stable patients with steady state serum creatinine concentration, CL_CR_, which is a surrogate marker of GFR, is higher in polytrauma patients than in other critically ill patients. In ICU patients, the drug monitoring must take into account the glomerular filtration rate. The measure of CL_CR _should be routinely proposed for PT patients in order to adjust dose regimen, especially for drugs with renal elimination (betalactams, ceftazidime, cefepime, piperacillin, vancomycin, aminoglycosides, and so on).

## Key messages

• In ICU patients with normal serum creatinine, CL_CR_, is higher in trauma than in non-trauma patients.

• The measure of CL_CR _should be proposed in routine for ICU patients in order to adjust dose regimen, especially for drugs with renal elimination.

• Age and trauma were the only factors independently correlated to CL_CR_.

• Glomerular filtration rate should be measured in ICU patient to detect renal filtration abnormalities.

• Serum creatinine is not a good marker for renal function estimation.

## Abbreviations

AKI: Acute Kidney Injury; CL_CR_: creatinine clearance; GFR: glomerular filtration rate; ICU: intensive care unit; NPT: non polytrauma patients; PT: polytrauma patients; sMDRD, Modification of Diet in Renal Disease index; SOFA: score, Sequential Organ Failure Assessment score.

## Competing interests

The authors declare that they have no competing interests.

## Authors' contributions

AJ and IT carried out the serum creatinine measurement and calibration. SR, AB and TS carried out the patients' inclusions. KA helped to draft the manuscript and reviewed the intellectual content. BG, SS, OF and KS participated in the design of the study and helped to draft the manuscript. JMC and VM conceived of the study, and participated in its design and coordination and helped to draft the manuscript and performed the statistical analysis. All authors read and approved the final manuscript.

## References

[B1] Conil JM Georges B Fourcade O Seguin T Lavit M Samii K Houin G Tack I Saivin S Assessment of renal function in clinical practice at the bedside of burn patients Br J Clin Pharmacol 2007 63 583 594 10.1111/j.1365-2125.2006.02807.x 17166188 PMC2000748

[B2] Loirat P Rohan J Baillet A Beaufils F David R Chapman A Increased glomerular filtration rate in patients with major burns and its effect on the pharmacokinetics of tobramycin N Engl J Med 1978 299 915 919 10.1056/NEJM197810262991703 692596

[B3] Conil JM Georges B Breden A Segonds C Lavit M Seguin T Coley N Samii K Chabanon G Houin G Saivin S Increased amikacin dosage requirements in burn patients receiving a once-daily regimen Int J Antimicrob Agents 2006 28 226 230 10.1016/j.ijantimicag.2006.04.015 16908121

[B4] Conil JM Georges B Lavit M Laguerre J Samii K Houin G Saivin S A population pharmacokinetic approach to ceftazidime use in burn patients: influence of glomerular filtration, gender and mechanical ventilation Br J Clin Pharmacol 2007 64 27 35 10.1111/j.1365-2125.2007.02857.x 17324245 PMC2000604

[B5] Conil JM Georges B Mimoz O Dieye E Ruiz S Cougot P Samii K Houin G Saivin S Influence of renal function on trough serum concentrations of piperacillin in intensive care unit patients Intensive Care Med 2006 32 2063 2066 10.1007/s00134-006-0421-1 17061021

[B6] Larsson R Bodemar G Kagedal B Walan A The effects of cimetidine (Tagamet) on renal function in patients with renal failure Acta Med Scand 1980 208 27 31 10.1111/j.0954-6820.1980.tb01145.x 7001858

[B7] Hull JH Hak LJ Koch GG Wargin WA Chi SL Mattocks AM Influence of range of renal function and liver disease on predictability of creatinine clearance Clin Pharmacol Ther 1981 29 516 521 10.1038/clpt.1981.72 7471619

[B8] Papadakis MA Arieff AI Unpredictability of clinical evaluation of renal function in cirrhosis. Prospective study Am J Med 1987 82 945 952 10.1016/0002-9343(87)90156-2 3578363

[B9] Ferreira FL Bota DP Bross A Melot C Vincent JL Serial evaluation of the SOFA score to predict outcome in critically ill patients JAMA 2001 286 1754 1758 10.1001/jama.286.14.1754 11594901

[B10] Antonelli M Moreno R Vincent JL Sprung CL Mendoca A Passariello M Riccioni L Osborn J Application of SOFA score to trauma patients. Sequential Organ Failure Assessment Intensive Care Med 1999 25 389 394 10.1007/s001340050863 10342513

[B11] Vincent JL de Mendonca A Cantraine F Moreno R Takala J Suter PM Sprung CL Colardyn F Blecher S Use of the SOFA score to assess the incidence of organ dysfunction/failure in intensive care units: results of a multicenter, prospective study. Working group on "sepsis-related problems" of the European Society of Intensive Care Medicine Crit Care Med 1998 26 1793 1800 9824069 10.1097/00003246-199811000-00016

[B12] Froissart M Rossert J Jacquot C Paillard M Houillier P Predictive performance of the modification of diet in renal disease and Cockcroft-Gault equations for estimating renal function J Am Soc Nephrol 2005 16 763 773 10.1681/ASN.2004070549 15659562

[B13] Cockcroft DW Gault MH Prediction of creatinine clearance from serum creatinine Nephron 1976 16 31 41 10.1159/000180580 1244564

[B14] Robert S Zarowitz BJ Peterson EL Dumler F Predictability of creatinine clearance estimates in critically ill patients Crit Care Med 1993 21 1487 1495 10.1097/00003246-199310000-00016 8403957

[B15] Levey AS Bosch JP Lewis JB Greene T Rogers N Roth D A more accurate method to estimate glomerular filtration rate from serum creatinine: a new prediction equation. Modification of Diet in Renal Disease Study Group Ann Intern Med 1999 130 461 470 10075613 10.7326/0003-4819-130-6-199903160-00002

[B16] Hoste EA Damen J Vanholder RC Lameire NH Delanghe JR Van den Hauwe K Colardyn FA Assessment of renal function in recently admitted critically ill patients with normal serum creatinine Nephrol Dial Transplant 2005 20 747 753 10.1093/ndt/gfh707 15701668

[B17] Conil JM Georges B Lavit M Seguin T Tack I Samii K Chabanon G Houin G Saivin S Pharmacokinetics of ceftazidime and cefepime in burn patients: the importance of age and creatinine clearance Int J Clin Pharmacol Ther 2007 45 529 538 17966838 10.5414/cpp45529

[B18] Conil JM Favarel H Laguerre J Brouchet A Chabanon G Cazal L Bodnar M Rouge D Virenque C Costagliola M [Continuous administration of vancomycin in patients with severe burns] Presse Med 1994 23 1554 1558 7824489

[B19] Conil JM Georges B de Lussy A Khachman D Seguin T Ruiz S Cougot P Fourcade O Houin G Saivin S Ciprofloxacin use in critically ill patients: pharmacokinetic and pharmacodynamic approaches Int J Antimicrob Agents 2008 32 505 510 10.1016/j.ijantimicag.2008.05.019 18768301

[B20] O'Connell MB Dwinell AM Bannick-Mohrland SD Predictive performance of equations to estimate creatinine clearance in hospitalized elderly patients Ann Pharmacother 1992 26 627 635 1591419 10.1177/106002809202600503

[B21] Grotsch H Hajdu P Interference by the new antibiotic cefpirome and other cephalosporins in clinical laboratory tests, with special regard to the "Jaffe" reaction J Clin Chem Clin Biochem 1987 25 49 52 3559483

[B22] Doi K Yuen PS Eisner C Hu X Leelahavanichkul A Schnermann J Star RA Reduced production of creatinine limits its use as marker of kidney injury in sepsis J Am Soc Nephrol 2009 20 1217 1221 10.1681/ASN.2008060617 19389851 PMC2689892

[B23] Macedo E Bouchard J Soroko SH Chertow GM Himmelfarb J Ikizler TA Paganini EP Mehta RL Fluid accumulation, recognition and staging of acute kidney injury in critically-ill patients Crit Care 2010 14 R82 10.1186/cc9004 20459609 PMC2911707

[B24] Hoen S Asehnoune K Brailly-Tabard S Mazoit JX Benhamou D Moine P Edouard AR Cortisol response to corticotropin stimulation in trauma patients: influence of hemorrhagic shock Anesthesiology 2002 97 807 813 10.1097/00000542-200210000-00010 12357144

[B25] Stevens LA Coresh J Greene T Levey AS Assessing kidney function--measured and estimated glomerular filtration rate N Engl J Med 2006 354 2473 2483 10.1056/NEJMra054415 16760447

[B26] Georges B Conil JM Seguin T Ruiz S Minville V Cougot P Decun JF Gonzalez H Houin G Fourcade O Saivin S Population pharmacokinetics of ceftazidime in intensive care unit patients: influence of glomerular filtration rate, mechanical ventilation, and reason for admission Antimicrob Agents Chemother 2009 53 4483 4489 10.1128/AAC.00430-09 19635962 PMC2764213

